# A Structural Model for the Ligand Binding of Pneumococcal Serotype 3 Capsular Polysaccharide-Specific Protective Antibodies

**DOI:** 10.1128/mBio.00800-21

**Published:** 2021-06-01

**Authors:** Ahmet Ozdilek, Jiachen Huang, Rachelle Babb, Amy V. Paschall, Dustin R. Middleton, Jeremy A. Duke, Liise-anne Pirofski, Jarrod J. Mousa, Fikri Y. Avci

**Affiliations:** a Department of Biochemistry and Molecular Biology, University of Georgia, Athens, Georgia, USA; b Center for Molecular Medicine, University of Georgia, Athens, Georgia, USA; c Department of Infectious Diseases, College of Veterinary Medicine, University of Georgia, Athens, Georgia, USA; d Center for Vaccines and Immunology, College of Veterinary Medicine, University of Georgia, Athens, Georgia, USA; e Division of Infectious Diseases, Department of Medicine, Albert Einstein College of Medicine and Montefiore Medical Center, Bronx, New York, USA; Brigham and Women's Hospital/Harvard Medical School

**Keywords:** carbohydrate antigens, capsular polysaccharide, monoclonal antibody, VH3 gene family, *Streptococcus pneumoniae*, conjugate vaccines, glycoconjugate vaccine, monoclonal antibodies

## Abstract

Capsular polysaccharides (CPSs) are major virulence factors that decorate the surfaces of many human bacterial pathogens. In their pure form or as glycoconjugate vaccines, CPSs are extensively used in vaccines deployed in clinical practice worldwide. However, our understanding of the structural requirements for interactions between CPSs and antibodies is limited. A longstanding model based on comprehensive observations of antibody repertoires binding to CPSs is that antibodies expressing heavy chain variable gene family 3 (VH3) predominate in these binding interactions in humans and VH3 homologs in mice. Toward understanding this highly conserved interaction, we generated a panel of mouse monoclonal antibodies (MAb) against Streptococcus pneumoniae serotype 3 CPS, determined an X-ray crystal structure of a protective MAb in complex with a hexasaccharide derived from enzymatic hydrolysis of the polysaccharide, and elucidated the structural requirements for this binding interaction. The crystal structure revealed a binding pocket containing aromatic side chains, suggesting the importance of hydrophobicity in the interaction. Through mutational analysis, we determined the amino acids that are critical in carbohydrate binding. Through elucidating the structural and functional properties of a panel of murine MAbs, we offer an explanation for the predominant use of the human VH3 gene family in antibodies against CPSs with implications in knowledge-based vaccine design.

## INTRODUCTION

Staphylococcus aureus (1), Neisseria meningitidis (2), Salmonella enterica serovar Typhi (3), and Streptococcus pneumoniae (4) are a few examples of major human pathogens that possess polysaccharide capsules (5). Such capsules play important roles in the virulence of bacteria (6), as they protect them from complement-mediated killing, bind and keep cationic antimicrobial peptides (CAMPs) at a distance from bacterial membranes (7, 8), prevent attachment and subsequent internalization by phagocytes, and prevent phagosome-lysosome fusion (9). Capsules cover the bacteria and act as a shield around them. Streptococcus pneumoniae, for example, is embedded in a thick CPS layer that isolates and protects the bacterium from immune responses (4). Since an immune response toward this protective barrier can render bacteria less virulent, capsular polysaccharides are utilized as antigens in many vaccines, such that immune responses are directed to these structures (10).

Raising antibodies against pathogenic organisms via vaccination has been a major form of protection (11). For pure polysaccharide vaccines and glycoconjugate vaccines, it is the capsular polysaccharide that is the desired target of antibody responses. Therefore, understanding the structural and biochemical requirements for how carbohydrate-specific antibodies bind to their ligands is of great importance for designing vaccines against these antigens. Previous studies have demonstrated that, in human antibodies, there is a biased usage of the VH3 gene family against carbohydrates, especially bacterial capsular polysaccharides, including capsular polysaccharides of Streptococcus pneumoniae type 23F, 8, 6B, and 3, Haemophilus influenzae type b, and Cryptococcus neoformans (12–21), but the preferred binding mechanism utilized by the VH3 gene family is not known. Another previous report described the binding of human VH3-encoded MAbs to pneumococcal type 3 polysaccharide Pn3P (22). Based on the findings of this study, a relationship with antibody-antigen interaction and the biological function of the antibody was proposed. We set up a study to investigate this mechanism using Streptococcus pneumoniae capsular polysaccharide 3, since serotype 3 is one of the leading serotypes causing complicated pneumococcal pneumonia (CPP) (23), otitis media in children (24), and invasive pneumococcal disease in adults and older children (25). Pn3P is an important virulence factor required for the bacterium to cause disease in human hosts (26). Enzymatic hydrolysis of the Pn3P on the bacterial surface reduces its virulence dramatically by eliminating the protective capsular shield and leaving bacteria defenseless against host immune clearance (27–29). Pn3P is a linear polysaccharide consisting of -3)β-d-Glc*p*A(1– 4)β-d-Glc*p*(1- disaccharide repeating units (30–32). Numerous studies have shown that current pneumococcal multivalent conjugate vaccines are less effective against serotype 3 than against other vaccine-included serotypes and incidence rates of serotype 3 continue to rise (33–39).

By elucidating the interactions between a protective mouse Pn3P-specific monoclonal antibody that is derived from a human VH3 homolog and its Pn3 hexasaccharide ligand, we show the hydrophobic amino acids within and around the antigen-binding site of the carbohydrate-specific antibodies can be critical for the binding. The structural data presented here may serve as a working hypothesis to explain the predominant use of the human VH3 gene family in carbohydrate-specific antibodies at a structural level. Structural and functional characterization of the antigenic determinants of capsular polysaccharides will enable the production of knowledge-based vaccine targets against pathogenic microorganisms, such as the highly virulent Streptococcus pneumoniae serotype 3.

## RESULTS

### V(D)J gene usage of Pn3P-specific monoclonal antibodies.

To elucidate the structural and functional properties of Pn3P-antibody interactions, we first generated five Pn3P-specific monoclonal antibodies (MAbs) (Table 1). Nucleotide sequences encoding each clone were obtained from cDNA, and V(D)J gene segments of each antibody were determined by using IgBLAST (Table 1) (40). Based on their shared V(D)J gene segment compositions, antibodies were classified as two groups, with MAbs 75.3 and 90.1 forming one group and MAbs 5.6, 25.1, and 64.4 forming the other. As the nucleotide sequences of the two groups are significantly different (Table 1), it is conceivable that these two groups were generated from multiple clones. However, the MAbs in each group may have originated from a single clone with the same germ line genes, but underwent different somatic hypermutations due to their highly similar sequences (Table 1). IgBLAST assigned a different D_H_ gene segment for MAb 5.6 compared to 25.1 and 64.4, possibly due to its single nucleotide mutation from the germ line (Fig. S1 in the supplemental material). MAbs 75.3 and 90.1 share identical heavy chain complementarity-determining region 1 (HCDR1), HCDR3, light chain CDR 2 (LCDR2), and LCDR3 sequences (Fig. S2). Among LCDRs, 75.3 and 90.1 have one and two amino acid changes compared to germ line genes, respectively, while their LCDR3s were identical with the germ line sequence (Fig. S2). MAbs 5.6, 25.1, and 64.4 use VH5 and share conserved D and J gene usage, as well as light chain usage with differences in CDRs of heavy and light chains due to somatic mutations (Fig. S2). All three MAbs have the same insertions in HCDR3. Amino acid sequences in all LCDRs of MAb 64.4 match with the germ line, while LCDRs of MAbs 5.6 and 25.1 have one and two amino acid changes, respectively (Fig. S2).

**TABLE 1:** V(D)J gene segments of Pn3P-specific mouse MAbs

mAb	V_H_	D_H_	J_H_	HCDR3	V_L_	J_L_	LCDR3
**75.3**	V_H_9-3-1	D_H_2-12 / D_H_2-2 / D_H_2-4	J_H_3	VRGNFTTGAWFTF	V_K_1-122	J_K_1	LQVTHVPWT
**90.1**	V_H_9-3-1	D_H_2-12 / D_H_2-2 / D_H_2-4	J_H_3	VRGNFTTGAWFTF	V_K_1-122	J_K_1	LQVTHVPWT
**5.6**	V_H_5-6-5	D_H_1-1	J_H_4	TRRVVVTKGGAMDY	V_K_4-55	J_K_4	QQWSTYPFT
**25.1**	V_H_5-6-5	D_H_2-12 / D_H_2-2 / D_H_2-7	J_H_4	TRRLVVTKGGAMDY	V_K_4-55	J_K_4	QQWSYYPFT
**64.4**	V_H_5-6-5	D_H_2-12 / D_H_2-2 / D_H_2-7	J_H_4	ARRVVVTRGGAMDY	V_K_4-55	J_K_4	QQWSSYPFT

10.1128/mBio.00800-21.1 FIG S1 Comparison of nucleotide sequences of MAb heavy chain V-D junctions, D gene segments, and D-J junctions. Nucleotides that differ among the MAbs in the same group are highlighted in yellow. Download FIG S1, PDF file, 0.4 MB. Copyright © 2021 Ozdilek et al. 2021 Ozdilek et al. https://creativecommons.org/licenses/by/4.0/ This content is distributed under the terms of the Creative Commons Attribution 4.0 International license.

10.1128/mBio.00800-21.2 FIG S2 Comparison of the amino acid sequences of MAb variable domains with their respective germline sequences. Amino acid sequences of MAbs were numbered according to IMGT and compared with their respective germline sequences. CDRs are indicated with red boxes and amino acids that differ from germline are highlighted. Amino acids tested and determined to be critical for ligand binding are indicated with arrows. Download FIG S2, PDF file, 0.5 MB. Copyright © 2021 Ozdilek et al. 2021 Ozdilek et al. https://creativecommons.org/licenses/by/4.0/ This content is distributed under the terms of the Creative Commons Attribution 4.0 International license.

### Pn3P-specific monoclonal antibodies have higher affinities to hexasaccharide than tetrasaccharide.

**FIG 1:**
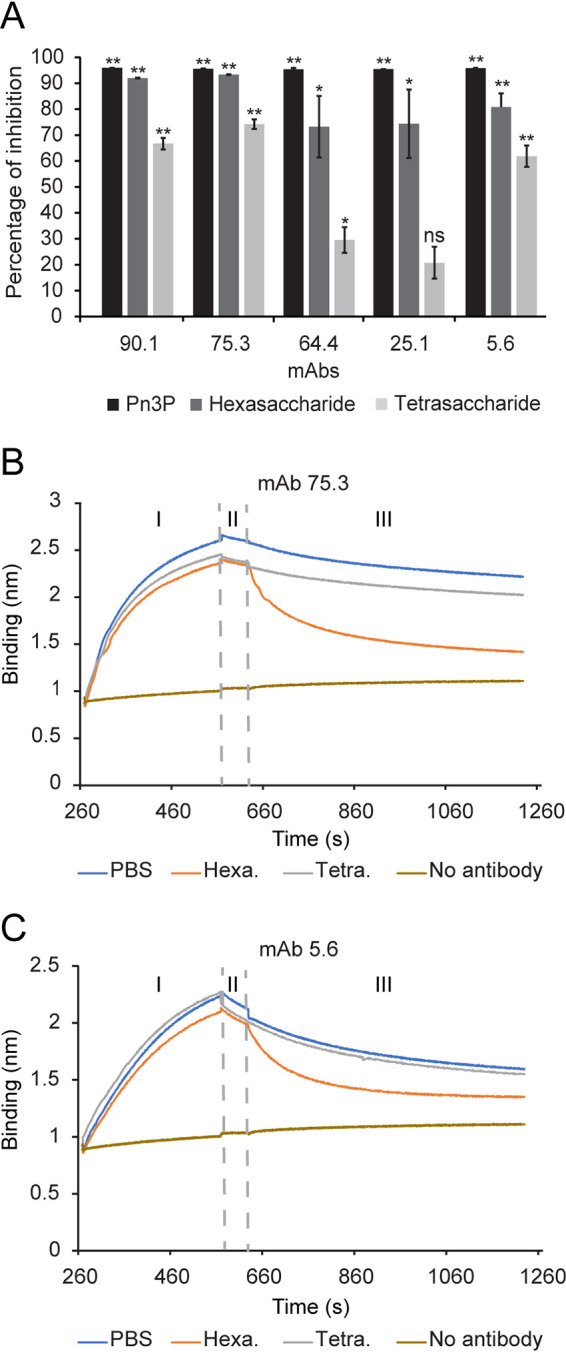
(A) Pn3P-specific monoclonal antibodies can bind to Pn3 hexasaccharide and tetrasaccharide. ELISA plates were coated with Pn3P-HSA and MAbs were premixed with Pn3P, Pn3 hexasaccharide, Pn3 tetrasaccharide, or PBS and then added to the wells. Both oligosaccharides inhibited antibody binding to polysaccharide, with the inhibition by Pn3 hexasaccharide significantly stronger. The PBS control group was considered to have 100% binding, against which the experimental groups were normalized. Using a two-tailed Student’s *t* test, statistical significance was determined compared to no- inhibitor wells; **, *P* < 0.01; *, *P* < 0.05; ns, not significant. (B and C) Pn3 hexasaccharide can dissociate Pn3P-bound antibodies, whereas tetrasaccharide cannot. In a BLI experiment, avidin sensors loaded with biotinylated Pn3P were incubated with MAb 75.3 (B) or MAb 5.6 (C) for 300 s (I). After incubation in BLI assay buffer with no competitor for 60 s (II), sensors were incubated in 50 μg/ml of either tetrasaccharide or hexasaccharide to compete with the polysaccharide for 600 s (III).

The antibody binding properties of Pn3 hexasaccharide and tetrasaccharide were assessed by a competition enzyme-linked immunosorbent assay (ELISA). Each monoclonal antibody was first incubated with Pn3 hexasaccharide, Pn3 tetrasaccharide, or Pn3P and then added to the ELISA plate coated with Pn3P-human serum albumin (HSA). Compared to samples with no competitors, hexasaccharide and tetrasaccharide significantly reduced Pn3P binding of all or four out of the five antibodies, respectively (Fig. 1A). For all antibodies, hexasaccharide displayed a higher inhibition activity than the tetrasaccharide. We then tested whether the Pn3 oligosaccharides could compete out the antibody-bound polysaccharide by biolayer interferometry (BLI). We tested one monoclonal antibody from each group shown in Table 1, MAbs 75.3 and 5.6, due to their strong oligosaccharide binding in ELISA (Fig. 1A). In this experiment, biotinylated Pn3P loaded to streptavidin-coated sensors were first associated with MAbs 75.3 and 5.6. Sensors were then placed in BLI assay buffer containing Pn3 hexasaccharide or tetrasaccharide. Tetrasaccharide was not able to release antibodies from the sensor, while hexasaccharide could, indicating a stronger binding between hexasaccharide and the antibodies (Fig. 1B and C).

### MAb 5.6 Fab binds to the trisaccharide region within the Pn3 hexasaccharide.

**FIG 2:**
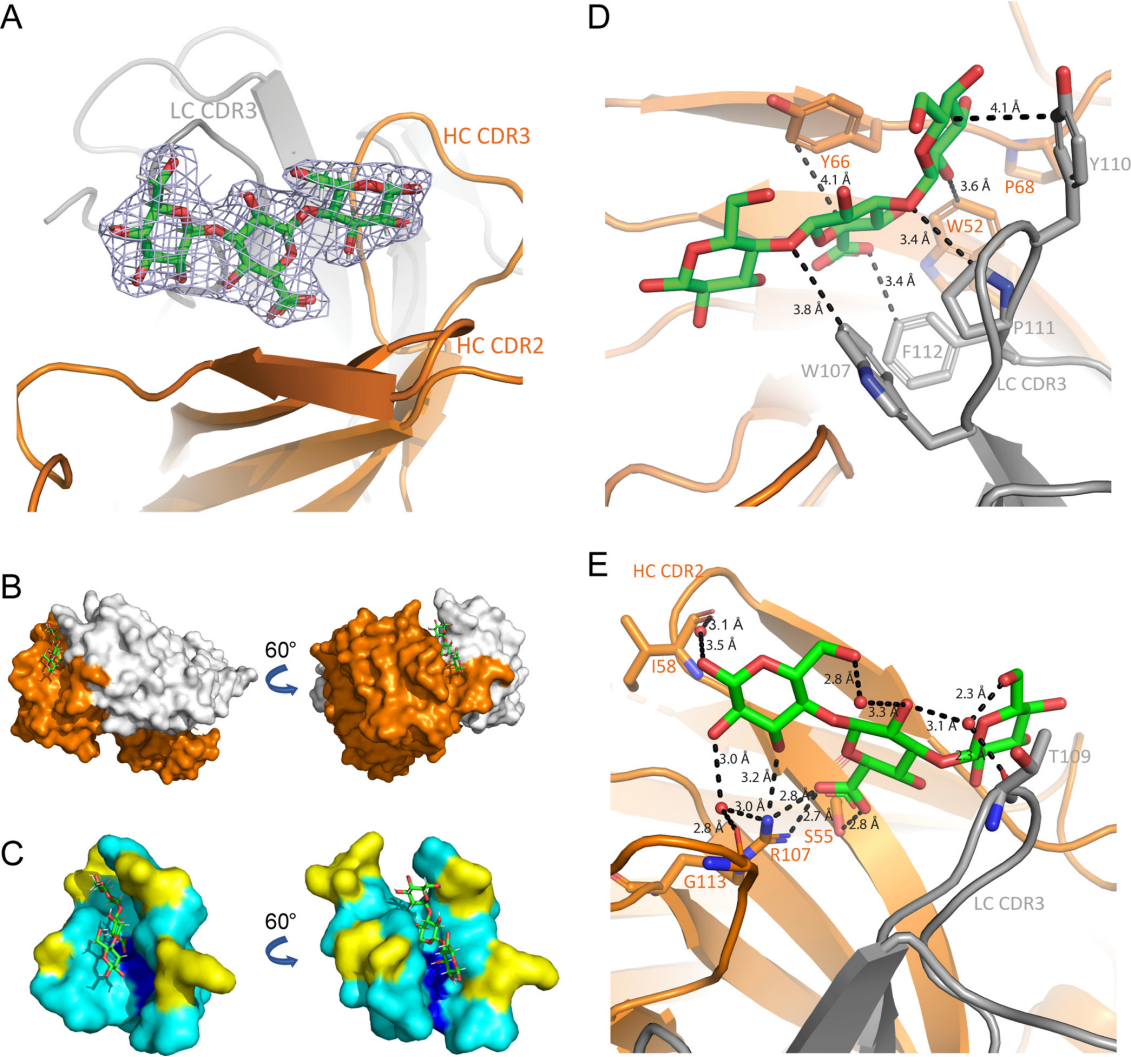
Crystal structure of Pn3 trisaccharide bound to MAb 5.6 Fab. (A) The crystal structure of MAb 5.6 Fab and the trisaccharide portion of Pn3 hexasaccharide was solved at 2.3 Å resolution. The trisaccharide Glc-GlcA-Glc fits to the electron density observed at the antigen binding site of the MAb 5.6 Fab and is depicted with sticks. Heavy and light chains are colored as orange and gray, respectively. (B) Fab is depicted with surfaces and the trisaccharide with sticks. Heavy and light chains are colored as orange and gray, respectively. (C) Antigen-binding site of the Fab is depicted with surfaces and the trisaccharide with sticks. Hydrophobic residues on the binding groove are colored as cyan, asparagine and serine residues involved in electrostatic and hydrogen binding interactions with the trisaccharide are colored as blue, and other residues as yellow. (D) There are seven aromatic residues around the ligand. The GlcA residue of the trisaccharide forms an aromatic stacking with Y66 of the heavy chain and a probable aromatic stacking with Y110 of the light chain. (E) The trisaccharide forms a hydrogen bond and electrostatic interactions with the side chains of S55 and R107. Three water bridges are formed between the trisaccharide and backbone atoms of G113, T109, and I58. The side chain of R107 is also involved in the water bridge formed between the trisaccharide and G113.

The human homolog of the MAb 5.6 heavy chain variable gene segment was determined to be a VH3 gene family member by IgBLAST (Fig. S3) (40). V_H_ gene segments of MAbs 25.1 and 64.4 are also homologous to the human VH3 gene family, whereas V_H_ gene segments of MAbs 75.3 and 90.1 are homologous to the human VH7 gene family. To elucidate the interactions between MAb 5.6 and its ligand at the structural level, we cocrystallized the antigen-binding fragment (Fab) region of MAb 5.6 with Pn3 hexasaccharide and the structure of the resulting complex was solved at 2.3 Å resolution (Fig. 2). In this cocrystal structure, an electron density of the internal trisaccharide residue Glc-GlcA-Glc was detected due to flexibility of unbound regions of the Pn3 hexasaccharide (Fig. 2A and B). The trisaccharide observed on the Fab is located in a boat-like groove, which contains arginine and serine residues at the bottom to form electrostatic and hydrogen bonding interactions with the ligand, respectively. Interestingly, the sides of the groove were predominantly populated with hydrophobic amino acids (Fig. 2C). There are seven aromatic side chains in the pocket around the ligand, two of which (Y66 of the heavy chain and Y110 of the light chain) potentially facilitate the ligand binding through aromatic stackings (Fig. 2D) (41, 42). We also observed three water bridges between the ligand and backbones of the I58, T109, and G113 residues and the side chain of the R107 residue in the groove (Fig. 2E).

10.1128/mBio.00800-21.3 FIG S3 Comparison of the amino acid sequence of MAb 5.6 heavy chain variable gene segment with its human homolog. CDRs are indicated with red boxes and amino acids that match between two sequences are highlighted. Download FIG S3, PDF file, 0.4 MB. Copyright © 2021 Ozdilek et al. 2021 Ozdilek et al. https://creativecommons.org/licenses/by/4.0/ This content is distributed under the terms of the Creative Commons Attribution 4.0 International license.

### Hydrophobic amino acids contribute to carbohydrate binding.

**FIG 3:**
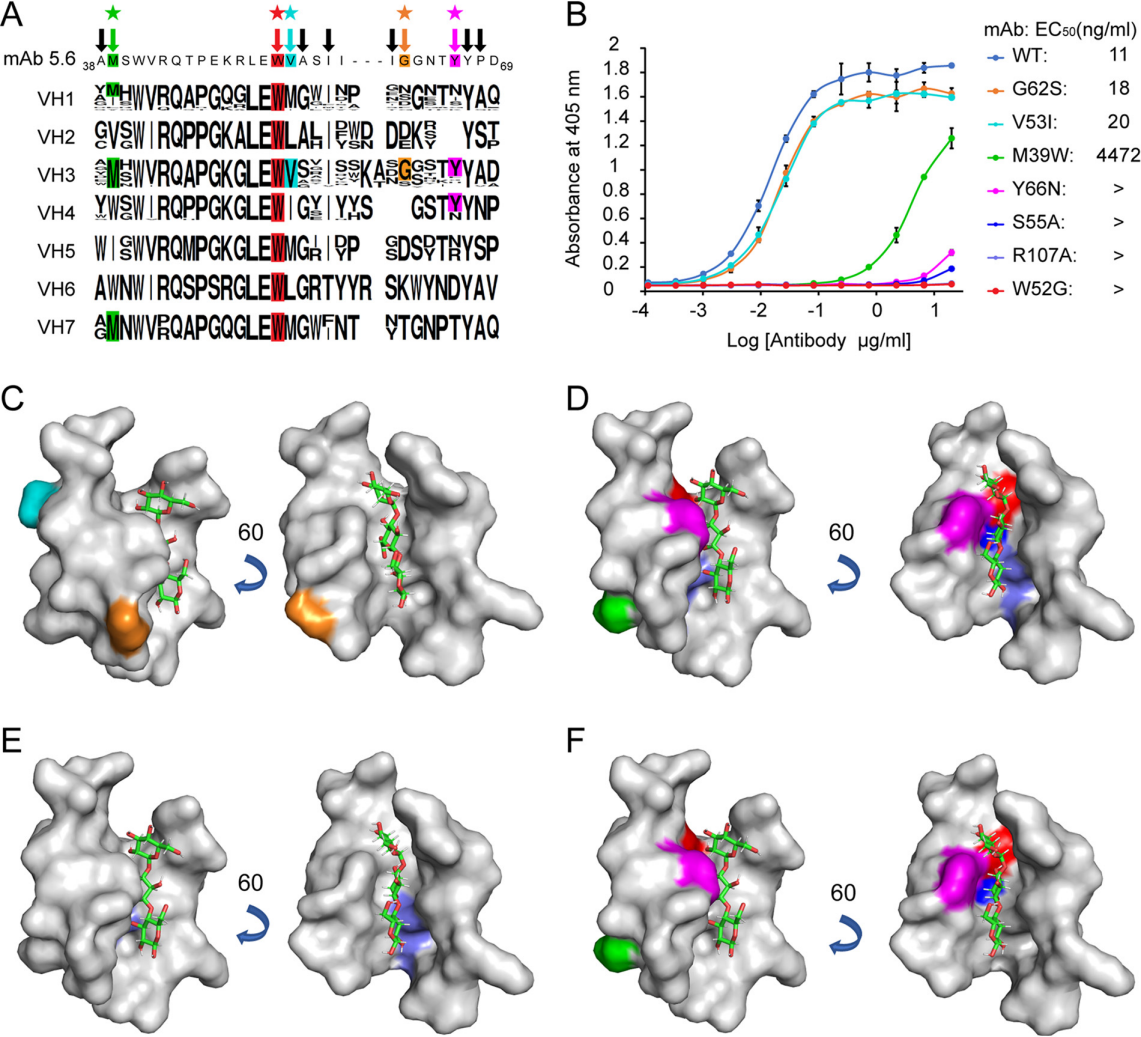
Hydrophobic amino acids of the VH3 gene family contribute to carbohydrate binding. (A) Hydrophobic amino acids of MAb 5.6 heavy chain variable gene segment located around the trisaccharide are indicated by arrows. Sequence logos for seven human VH gene families were generated to show conserved amino acids within the families. Amino acids indicated with stars were mutated to assess their potential roles in ligand binding. (B) In an ELISA, ligand binding of wild type and MAb 5.6 single site mutants M39W, V53I, G62S, Y66N, W52G, S55A, and R107A were evaluated; “>” indicates no signal was observed below 2,222 ng/ml. (C) Mutations on V53 (cyan) and G62 (orange) did not alter the ligand binding. (D) Mutations on M39 (green), Y66 (purple), W52 (red), R107 (light blue), and S55 (blue) reduced the ligand binding of 5.6 MAb. (E) R107 (light blue) in HCDR3 is essential for ligand binding. (F) M39 (green), Y66 (purple), W52 (red), and S55 (blue) are in framework regions and essential for ligand binding.

To understand why the VH3 gene family is predominantly used in polysaccharide-specific antibodies, amino acid sequences of all human heavy chain variable domains were compared with the hydrophobic amino acids observed around the ligand in the crystal structure (Fig. 3A). M39, V53, G62, and Y66 were determined to be present exclusively in VH3 gene family. These amino acids and the two amino acids that are involved in hydrogen bonding and electrostatic interactions with the ligand, S55 and R107, were mutated to elucidate their contributions to the ligand binding. W52, a common residue among all human VH subgroups (Fig. 3A), was also selected for the mutation analysis, since it could be a major contributor to the hydrophobicity of the groove. M39, W52, V53, and S55 are located in the framework region 2 (FR2); G62 in the CDR2; Y66 in the FR3; and R107 in the CDR3 of the heavy chain. Antibodies with the following mutations were expressed in HEK293 cells and their binding to Pn3P were determined in ELISA: M39W, V53I, G62S, Y66N, W52G, S55A, and R107A. While mutations on G62 and V53 did not affect binding significantly, mutations on S55, R107, M39, W52, and Y66 dramatically reduced binding (Fig. 3B to D). Among the five amino acids that are essential for ligand binding, only one of them, R107, is within a CDR, others belong to framework regions (Fig. 3E and F, Fig. S2).

### Pn3P-specific monoclonal antibodies are protective.

**FIG 4:**
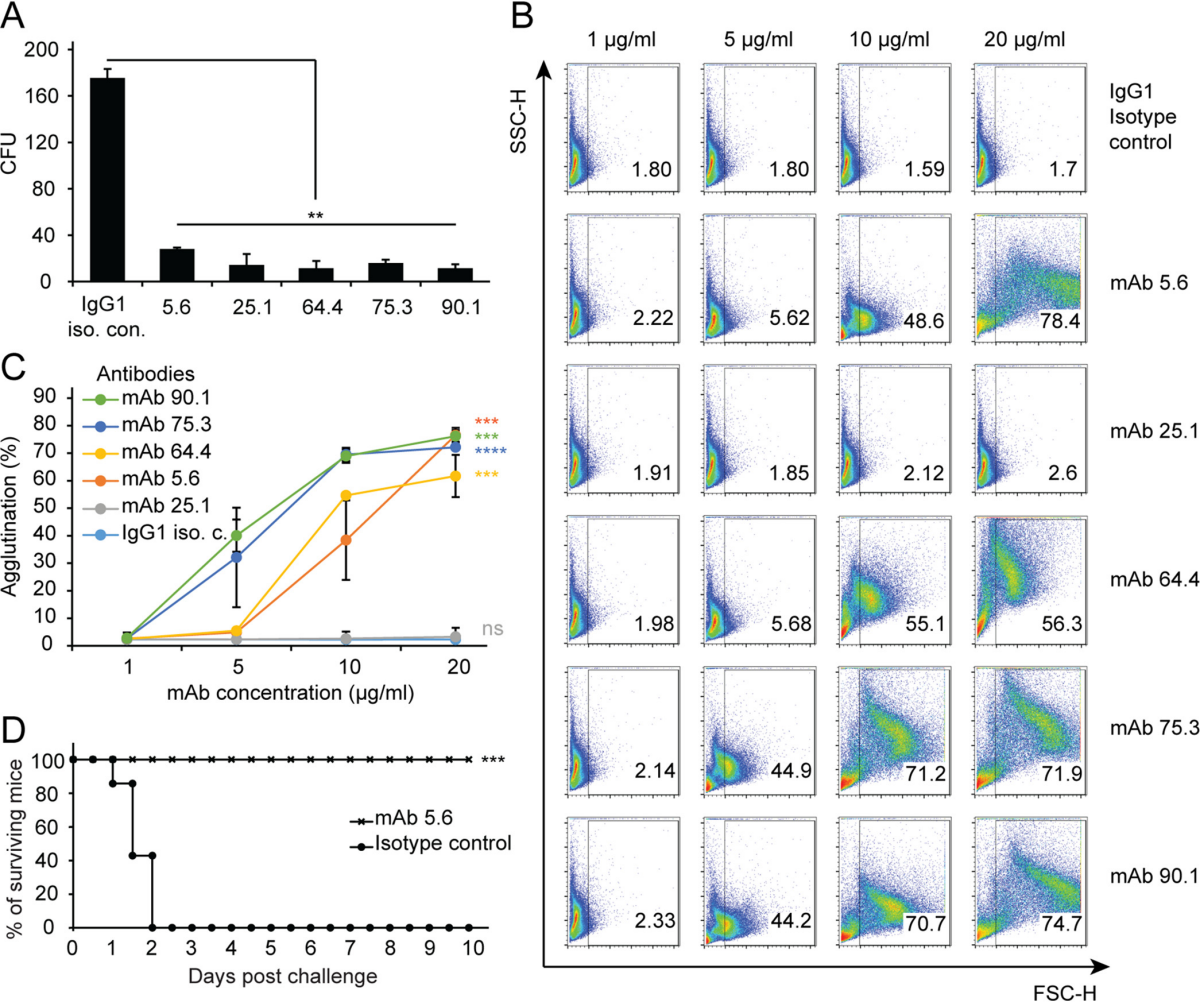
Pn3P-specific MAbs are protective. (A) MAbs were tested in a standard OPKA using differentiated HL-60 cells. The Spn3 WU2 strain was opsonophagocytosed with antibodies and HL-60 cells and plated onto blood agar plates to count CFU. Data are averages of duplicate samples. Statistical significance was determined with the two-tailed Student’s *t* test; **, *P* < 0.01. (B and C) The Spn3 B2 strain was incubated with increasing concentrations of various MAbs and nonspecific mouse IgG1 (control) and analyzed by flow cytometry. (B) Representative fluorescence-activated cell sorting (FACS) dot plots show the percentage of agglutination for all MAbs and isotype control (IgG1) at various concentrations. (C) Graph shows the percentage of agglutination for all samples with increasing concentrations of MAbs (1 to 20 μg/ml). Results represent 2 independent experiments. Statistical significance was determined with the two-tailed Student’s *t* test; ***, *P* < 0.001; ****, *P* < 0.0001. (D) Following i.p. injection of MAb 5.6 or its isotype control, two groups of mice were challenged with lethal dose of Spn3 WU2 strain. Graph shows the percentage of surviving mice in each group in a 10-day period. Statistical significance was determined with the logrank (Mantel-Cox) test; ***, *P* < 0.001.

We functionally characterized the Pn3P-specific monoclonal antibodies in an *in vitro* opsonophagocytosis killing assay (OPKA), since antibody-mediated opsonophagocytosis is critical for the clearance of Spn3 (43). All five MAbs induced phagocytosis of Spn3 (Fig. 4A). Pn3P-specific monoclonal antibodies were then evaluated for their ability to induce agglutination, since agglutination induced by antibodies is a protective mechanism through which colonization of bacteria is prevented (44). All MAbs, except 25.1, induced agglutination *in vitro* (Fig. 4B and C). We also tested MAb 5.6 in an *in vivo* infection experiment. Two groups of mice were injected intraperitoneally with MAb 5.6 or IgG1 isotype control and were then challenged with the Spn3 WU2 strain. Passive immunization with MAb 5.6 protected 100% of mice from lethal challenge, while none of the control group survived. (Fig. 4D). These results indicate protective abilities of MAbs both *in vitro* and *in vivo*.

## DISCUSSION

The human VH3 gene family is utilized preferentially in antibodies that bind to polysaccharide antigens (12–20, 45). Toward understanding this conserved interaction, we studied the carbohydrate-binding properties of the mouse MAb 5.6, the heavy chain variable gene segment of which belongs to the murine VH5 family that is homologous to the human VH3 gene family (Fig. S3). Observing a hydrophobic groove in the crystal structure of its ligand-binding site (Fig. 2), we hypothesized that this hydrophobic groove is important for carbohydrate binding as a common feature of the VH3 gene family, rendering human VH3 the main gene family utilized in polysaccharide-specific antibodies. Based on the crystal structure of the MAb 5.6 and the comparison of its sequence with human heavy chain variable gene segments, we determined the amino acids that may have roles in carbohydrate binding and exchanged them with amino acids that are prevalent in those positions of other human VH gene families. For this purpose, we chose M39W, V53I, G62S, Y66N, W52G, S55A, and R107A mutations to evaluate the ligand binding of MAb 5.6. Among M39, V53, and G62, which are spatially distant from the ligand, only the M39W mutation decreased the binding of MAb 5.6 to its ligand. Tryptophan occupies this position in the VH4 gene family. Since the side chain of M39 is embedded in the protein, replacing it with an aromatic group may have resulted in a structural change in the variable domain to impact the ligand binding. V53 and G62 are not in the hydrophobic groove of MAb 5.6 (Fig. 3C), however, these residues are exclusively present in the VH3 gene family (Fig. 3A). V53I and G62S mutations did not alter the affinity of MAb 5.6 to its ligand; however, they may contribute to ligand binding of other CPS-specific VH3 gene family antibodies. The W52G mutation dramatically reduced binding, indicating the importance of not only hydrophobicity but also the aromatic side chains, since glycine could not compensate for the tryptophan. Aromatic side chains can interact with carbohydrates through CH/π interactions in a stacking geometry, where the aromatic and sugar units can be parallel or nonparallel (41, 42). Previous studies revealed the importance of carbohydrate/aromatic stacking in carbohydrate recognition of various proteins such as transporters (46), enzymes (47), and lectins (48). In our cocrystal structure, Y66 forms a parallel stacking with GlcA, which may be critical for keeping the ligand in that position in the groove (Fig. 2D). In addition, Y110 on the light chain may be forming another aromatic stacking with the ligand, as rings that are not parallel to each other can also form aromatic stacking, such as T shaped stacking (Fig. 2D) (41, 42, 49). Residues 107W, 111P, and 112F on the light chain also contact with the ligand, and all of these remain the same as in the germ line sequence (Fig. 2D, Fig. S2). Among the five amino acids essential for ligand binding, four of them belong to framework regions (Fig. 3F), with only one amino acid, R107, in the HCDR3 region (Fig. 3E). R107 of HCDR3 forms the essential electrostatic interaction with the Pn3 oligosaccharide (Fig. 2E) that is required for the binding (Fig. 3B). Mutation on HCDR2 residue G62 residue, which is conserved in the VH3 gene family, did not impact ligand binding significantly (Fig. 3B).

In addition to the water bridges between the ligand and four amino acid residues in the groove (Fig. 2E), we postulate that water as the solvent has an important role in stabilizing the Ab-ligand complex. Through hydrophobic effect, entropy of structured water increases as it is released from the apposed hydrophobic surface by the ligand, which favors the formation of the complex thermodynamically (50). The role of hydrophobicity in ligand binding of carbohydrate-specific antibodies was proposed previously (51). MAb BR96, for example, binds to Le^y^ tetrasaccharide through a hydrophobic pocket composed mainly of aromatic residues, which were suggested to mediate the binding (52). S-20-4, a Vibrio cholerae lipopolysaccharide-specific MAb, is another example of a carbohydrate-specific antibody with a hydrophobic pocket (53).

Carbohydrate-binding antibodies are often found in their germ line forms (54–56). The potential role of the hydrophobic groove in the germ line antibodies may explain their carbohydrate binding without the need for somatic hypermutations. Antigen binding sites of germ line antibodies are more flexible than antibodies that underwent somatic hypermutations (57). When a hydrophilic carbohydrate antigen enters the groove and expels structured water molecules, the hydrogen bonds that will be established between the water molecules expelled and the water molecules that are outside the groove will stabilize antibody-carbohydrate binding.

Here, we also identified the antigenic determinants of Pn3P and the minimum length of saccharide for antibody binding through a series of ELISA and BLI experiments, as well as a cocrystal structure. In both ELISA and BLI experiments, Pn3 hexasaccharide had stronger interactions with the Pn3 monoclonal antibodies compared to the Pn3 tetrasaccharide. In light of our cocrystal structure of MAb 5.6 and the Pn3 hexasaccharide, hexasaccharides (with a Glc or GlcA reducing end) may contain two trisaccharide-binding motifs, whereas tetrasaccharides have only one. Compared to oligosaccharides, polysaccharide has the highest binding, as it has multiple binding motifs in its chain structure. In addition, avidity contributes to the polysaccharide binding of the antibodies. We were unable to generate a cocrystal of MAb 5.6 Fab and Pn3 tetrasaccharide, likely due to its weaker binding interaction with MAb 5.6 (Fig. 1). The solved structure indicates that MAb 5.6 can bind to Pn3 oligosaccharides as small as a trisaccharide. An explanation for why we see only the trisaccharide portion of the hexasaccharide is that the parts of the hexasaccharide that are not bound to the Fab do not occupy the same space in all crystallized molecules. Therefore, a useful electron density was not obtained for those unbound regions of the hexasaccharide. The structure of trisaccharide which has GlcA in the middle fitted the electron density best. While glycoconjugate vaccines have provided great health benefits in controlling bacterial diseases, their chemical conjugations have often been empirical, with weakly controlled conjugation chemistries resulting in poorly characterized, heterogeneous and variably immunogenic glycoconjugate vaccines (58). For example, S. pneumoniae remains among the deadliest infectious agents despite the global vaccination programs (59). Specifically, the serotype 3 is among the most virulent strains and, despite current vaccination programs, morbidity of the type 3 strain remains high, raising questions regarding the efficacy of this vaccine (60). Thus, deciphering the antigenic determinants for binding of antibodies that mediate robust protection is important for designing knowledge-based glycoconjugate vaccines that are optimized for their B and T cell activation to induce long-lasting and protective immunity.

## MATERIALS AND METHODS

### Mice.

Eight-week-old female BALB/c mice were purchased from Taconic Biosciences (Hudson, NY, USA) and used in monoclonal antibody generation and bacterial challenge experiments. Mice were kept in microisolator cages and handled under a laminar flow hood. All mouse experiments were in compliance with the University of Georgia Institutional Animal Care and Use Committee under an approved animal use protocol.

### Conjugation of Pn3P with proteins.

Purified Pn3P powder was purchased from ATCC. Pn3P was first depolymerized to an average molecular weight of 100 kDa and conjugated with keyhole limpet hemocyanin (KLH) (Calbiochem) or human serum albumin (HSA) through periodate oxidation followed by reductive amination according to our published protocols (61–63).

### Monoclonal antibody generation.

A female BALB/c mouse was immunized on days 0, 21, and 42 with 10 μg of Pn3P-KLH emulsified in Freund’s adjuvant (Thermo Scientific) by subcutaneous injection. The spleen was harvested 3 days after the final booster immunization. A total of 10^8^ splenocytes were fused with 2 × 10^7^ Sp2/0 myeloma cells. Hybridomas were selected in complete medium (20% fetal bovine serum [FBS]) supplemented with sodium hypoxanthine (5 mM), aminopterin (20 μM), and thymidine (0.8 mM), (HAT, Thermo Fisher). Once colonies started to emerge, the presence of Pn3P-specific antibodies in the medium was tested in ELISAs with Pn3P-HSA-coated plates to exclude KLH-specific antibody responses. Colonies with positive results underwent limited dilutions to obtain single colonies in complete medium with 10% FBS and 1× HT supplement (Thermo Fisher). After confirming the presence of anti-Pn3P antibodies in their media, monoclonal cells were grown in regular complete medium with 10% FBS. Before purifying antibodies from media, cells were transferred to serum-free hybridoma medium (Thermo Fisher). Secreted antibodies in the medium were purified by protein G beads.

### Nucleic acid sequence analysis of variable regions and germ line gene assignments.

To determine the V(D)J gene segments of the five monoclonal antibodies, total RNA was isolated from hybridoma cells using the E.Z.N.A. MicroElute total RNA kit (Omega). From purified RNA, first strand cDNA synthesis was performed using iScript cDNA synthesis kit (Bio-Rad) according to the manufacturer’s instructions. To amplify the genes of heavy and light chains, primer sets described previously (64) were used and amplified gene fragments were sequenced by Eurofins Genomics, USA. Mouse monoclonal antibody germ line genes and their human homologs were determined using the sequence analysis tool IgBLAST (40).

### Oligosaccharide generation by enzymatic hydrolysis.

Pn3P powder (20 mg) was incubated with 0.5 mg of Pn3Pase (31) at 37°C for 48 h. The reaction was stopped by heating at 100°C for 5 min and loaded onto a 120 ml Superdex 30 column (GE). Products were separated in phosphate-buffered saline (PBS) with a flow rate of 1 ml/min and monitored by refractive index and absorbance at 205 nm. Fractions (0.5 ml) were collected, and oligosaccharide peaks were combined and desalted into water on a 30-ml packed fine P2 column (Bio-Rad). Desalted oligosaccharides were lyophilized, and hexasaccharide and tetrasaccharide identities were confirmed by matrix-assisted laser desorption ionization–time of flight mass spectrometry (MALDI-TOF MS) (31).

### Enzyme-linked immunosorbent assay.

Plates were coated with 1 μg Pn3P-HSA/ml in carbonate buffer at 4°C overnight. Unoccupied sites were blocked with 1% bovine serum albumin in PBS. Serial dilutions of wild-type or mutated antibodies were applied to the wells and incubated for 2 h at room temperature (RT). For the competition ELISA, before being added to the ELISA plate, antibodies were incubated with 50 μg/ml Pn3P, hexasaccharide, or tetrasaccharide for 2 h at RT. Goat anti-mouse IgG-HRP (horseradish peroxidase) (BioLegend) was used as a secondary antibody. Colorimetric measurements were taken after adding 3,3′,5,5′-tetramethylbenzidine (TMB, BioLegend) to develop color and 2 N sulfuric acid to cease the reaction.

### Biotinylation of Pn3P.

To generate biotinylated Pn3P, 2 mg of Pn3P was first dissolved in 0.5 ml of 0.1 M sodium borate buffer (pH 5.4). Then, 12 mg of hydrazide biotin was dissolved in 0.1 ml dimethyl sulfoxide (DMSO) and added to the polysaccharide containing solution, after which 1.5 mg of 1-ethyl-3-(3-dimethylaminopropyl) carbodiimide (EDC) was added and the solution was mixed at 25°C for 3 h. The product was purified using a Superdex 200 size exclusion column.

### Biolayer interferometry.

Whether hexasaccharides or tetrasaccharides can dissociate antibodies that are already bound to polysaccharides was determined by biolayer interferometry (BLI) using an OCTETRED384 system (ForteBio). The BLI assay buffer consisted of 1% bovine serum albumin (BSA) in 0.05% Tween 20 in PBS (PBST). After binding of 20 μg/ml biotinylated Pn3P to streptavidin biosensors for 150 s, 10 μg/ml IgG variants were associated (300 s) with the polysaccharide. In the dissociation step, after biosensors were incubated in BLI assay buffer that did not have any competitor (60 s), they were incubated in BLI assay buffers containing 50 μg/ml tetrasaccharides or hexasaccharides to compete with the polysaccharide.

### Crystallization and structure determination.

MAb 5.6 Fab purified from ficin digestion was concentrated to 15 mg/ml, mixed with Pn3 hexasaccharide in 1:5 molar ratio, and concentrated to 13 mg/ml. Crystallization trials were prepared on a TTP LabTech Mosquito Robot in sitting-drop MRC-2 plates (Hampton Research) using several commercially available crystallization screens. Crystals were obtained in the Crystal Screen HT (Hampton Research) in condition H10 (0.1 M sodium chloride, 0.1 M BICINE [pH 9.0], 20% vol/vol polyethylene glycol monomethyl ether 550). Crystals were harvested and cryoprotected with 30% glycerol in the mother liquor before being flash frozen in liquid nitrogen. X-ray diffraction data were collected at the Advanced Photon Source SER-CAT beamline 21-ID-D. Data were indexed and scaled using XDS. A molecular replacement solution was obtained in Phaser based on the mouse Fab structure (PDB 5XJM). The crystal structure was completed by manually building in COOT followed by subsequent rounds of manual rebuilding and refinement in Phenix. The data collection and refinement statistics are shown in Table S1 in the supplemental material.

10.1128/mBio.00800-21.4 TABLE S1 Data collection and refinement statistics for MAb 5.6 Fab-Pn3 hexasaccharide. Statistics for the highest-resolution shell are shown in parentheses. Download Table S1, DOCX file, 0.01 MB. Copyright © 2021 Ozdilek et al. 2021 Ozdilek et al. https://creativecommons.org/licenses/by/4.0/ This content is distributed under the terms of the Creative Commons Attribution 4.0 International license.

### Recombinant antibody expression.

Genes encoding heavy and light chains of MAb 5.6 were inserted into a mammalian expression vector separately. For ease of purification, a histidine tag was added to the C terminus of the heavy chain. Mutated heavy chain genes were generated through site-directed mutagenesis. Human embryonic kidney cells (HEK 293F) were cultured in FreeStyle 293 expression medium (Thermo Fisher Scientific). Cells were transiently transfected with 1 μg of each plasmid/ml and 9 μg polyethylenimine/ml (PEI, Polysciences). On day 6, culture medium was harvested, and antibodies were purified using a Ni-NTA affinity column.

### Bacterial strains and growth conditions.

S. pneumoniae serotype 3 (Spn3) strain WU2 (generous gift from Moon Nahm at the University of Alabama at Birmingham) was cultured aerobically at 37°C on tryptic soy agar with 5% sheep blood, or in Todd-Hewitt broth supplemented with 0.5% yeast extract (BD Biosciences) without shaking. Spn3 isolate B2, a strain isolated in the Montefiore Medical Center clinical microbiology laboratory (Albert Einstein College of Medicine IRB protocol 2014-4035) was grown in the same conditions as strain WU2.

### Opsonophagocytic killing assay.

An opsonophagocytic killing assay (OPKA) was performed as described previously (43), as adapted from an earlier protocol with modifications (65). WU2 stocks were incubated in duplicate wells in a 96-well round-bottom plate for 1 h at 37°C with the indicated antibodies (10 μg/100 μl/well) in opsonization buffer B (OBB; sterile 1× PBS with Ca^2+^/Mg^2+^, 0.1% gelatin, and 5% heat-inactivated FetalClone), with an IgG1 antibody serving as a control. Cells of the human promyelocytic leukemia cell line HL-60 (ATCC) were cultured in RPMI with 10% heat-inactivated FetalClone and 1% l-glutamine. HL-60 cells were differentiated using 0.6% *N*,*N*-dimethylformamide (DMF) for 3 days before performing the OPKA assay, harvested, and resuspended in OBB. Baby rabbit complement (Pel-Freez) was added to HL-60 cells at a 1:5 final volume. The HL-60-complement mixture was added to the bacteria at 5 × 10^5^ cells/well. The final reaction mixtures were incubated at 37°C for 1 h with shaking. The reactions were stopped by incubating the samples on ice for approximately 20 min. Then, 10 μl of each reaction mixture in duplicate was diluted to a final volume of 50 μl and plated onto blood agar plates. Plates were incubated overnight at 30°C and CFU counted the next day.

### *In vitro* agglutination assay.

Antibodies were tested for their ability to agglutinate Spn3 by flow cytometry as described (66, 67). Spn3, B2 strain (1 × 10^5^ CFU), was incubated in duplicate (total volume 50 μl) with increasing concentrations of various MAbs and a negative-control mouse IgG1 (Southern Biotech) diluted in PBS for 1 h at 37°C in a 96-well round-bottom plate. Cells were then fixed with 1% paraformaldehyde and analyzed by flow cytometry. Bacteria were gated based on forward scatter (FSC) and sideward scatter (SSC) (referring to cell size and granularity) to determine percentage agglutination.

### Murine sepsis challenge.

Groups of 7 unanesthetized mice were injected intraperitoneally (i.p.) with 20 μg of antibodies or a mouse IgG1 isotype control (BioLegend). An hour later, mice were injected i.p. with 1 × 10^4^ CFU of mid-log-phase WU2 strain bacteria. Animals were monitored every 12 h and the ones displaying morbidity criteria according to our IACUC-approved animal protocol were euthanized at a humane endpoint.

### Data availability.

Crystal structure data for the complex of the Fab region of MAb 5.6 and Pn3 hexasaccharide is available at https://www.rcsb.org/structure/7JVD.

## References

[1] O'Riordan K, Lee JC. 2004. Staphylococcus aureus capsular polysaccharides. Clin Microbiol Rev 17:218–234. doi:10.1128/cmr.17.1.218-234.2004. 14726462 PMC321462

[2] Harrison OB, Claus H, Jiang Y, Bennett JS, Bratcher HB, Jolley KA, Corton C, Care R, Poolman JT, Zollinger WD, Frasch CE, Stephens DS, Feavers I, Frosch M, Parkhill J, Vogel U, Quail MA, Bentley SD, Maiden MC. 2013. Description and nomenclature of Neisseria meningitidis capsule locus. Emerg Infect Dis 19:566–573. doi:10.3201/eid1904.111799. 23628376 PMC3647402

[3] Pickard D, Wain J, Baker S, Line A, Chohan S, Fookes M, Barron A, Gaora PO, Chabalgoity JA, Thanky N, Scholes C, Thomson N, Quail M, Parkhill J, Dougan G. 2003. Composition, acquisition, and distribution of the Vi exopolysaccharide-encoding Salmonella enterica pathogenicity island SPI-7. J Bacteriol 185:5055–5065. doi:10.1128/jb.185.17.5055-5065.2003. 12923078 PMC180996

[4] Yother J. 2011. Capsules of Streptococcus pneumoniae and other bacteria: paradigms for polysaccharide biosynthesis and regulation. Annu Rev Microbiol 65:563–581. doi:10.1146/annurev.micro.62.081307.162944. 21721938

[5] Zhensong W, Jing-Ren Z. 2015. Bacterial capsules, p 33–53. *In* Tang Y-W, Liu D, Schwartzman J, Sussman M, Poxton I (ed), Molecular Medical Microbiology, 2nd ed. Academic Press, Elsevier, Ltd.

[6] Willis LM, Whitfield C. 2013. Structure, biosynthesis, and function of bacterial capsular polysaccharides synthesized by ABC transporter-dependent pathways. Carbohydr Res 378:35–44. doi:10.1016/j.carres.2013.05.007. 23746650

[7] Campos MA, Vargas MA, Regueiro V, Llompart CM, Alberti S, Bengoechea JA. 2004. Capsule polysaccharide mediates bacterial resistance to antimicrobial peptides. Infect Immun 72:7107–7114. doi:10.1128/IAI.72.12.7107-7114.2004. 15557634 PMC529140

[8] Spinosa MR, Progida C, Tala A, Cogli L, Alifano P, Bucci C. 2007. The Neisseria meningitidis capsule is important for intracellular survival in human cells. Infect Immun 75:3594–3603. doi:10.1128/IAI.01945-06. 17470547 PMC1932921

[9] Nikulin J, Panzner U, Frosch M, Schubert-Unkmeir A. 2006. Intracellular survival and replication of Neisseria meningitidis in human brain microvascular endothelial cells. Int J Med Microbiol 296:553–558. doi:10.1016/j.ijmm.2006.06.006. 17010667

[10] Avci F, Berti F, Dull P, Hennessey J, Pavliak V, Prasad AK, Vann W, Wacker M, Marcq O. 2019. Glycoconjugates: what it would take to master these well-known yet little-understood immunogens for vaccine development. mSphere 4:e00520-19. doi:10.1128/mSphere.00520-19. 31554723 PMC6763769

[11] Pollard AJ, Bijker EM. 2021. A guide to vaccinology: from basic principles to new developments. Nat Rev Immunol 21:83–100. doi:10.1038/s41577-020-00479-7. 33353987 PMC7754704

[12] Chang Q, Zhong Z, Lees A, Pekna M, Pirofski L. 2002. Structure-function relationships for human antibodies to pneumococcal capsular polysaccharide from transgenic mice with human immunoglobulin Loci. Infect Immun 70:4977–4986. doi:10.1128/iai.70.9.4977-4986.2002. 12183544 PMC128266

[13] Scott MG, Zachau HG, Nahm MH. 1992. The human antibody V region repertoire to the type B capsular polysaccharide of Haemophilus influenzae. Int Rev Immunol 9:45–55. doi:10.3109/08830189209061782. 1282926

[14] Zhou J, Lottenbach KR, Barenkamp SJ, Reason DC. 2004. Somatic hypermutation and diverse immunoglobulin gene usage in the human antibody response to the capsular polysaccharide of Streptococcus pneumoniae Type 6B. Infect Immun 72:3505–3514. doi:10.1128/IAI.72.6.3505-3514.2004. 15155658 PMC415722

[15] Adderson EE, Shackelford PG, Quinn A, Carroll WL. 1991. Restricted Ig H chain V gene usage in the human antibody response to Haemophilus influenzae type b capsular polysaccharide. J Immunol 147:1667–1674. 1908880

[16] Zhou J, Lottenbach KR, Barenkamp SJ, Lucas AH, Reason DC. 2002. Recurrent variable region gene usage and somatic mutation in the human antibody response to the capsular polysaccharide of Streptococcus pneumoniae type 23F. Infect Immun 70:4083–4091. doi:10.1128/iai.70.8.4083-4091.2002. 12117915 PMC128163

[17] Pirofski L, Lui R, DeShaw M, Kressel AB, Zhong Z. 1995. Analysis of human monoclonal antibodies elicited by vaccination with a Cryptococcus neoformans glucuronoxylomannan capsular polysaccharide vaccine. Infect Immun 63:3005–3014. doi:10.1128/IAI.63.8.3005-3014.1995. 7622223 PMC173409

[18] Abadi J, Friedman J, Mageed RA, Jefferis R, Rodriguez-Barradas MC, Pirofski L. 1998. Human antibodies elicited by a pneumococcal vaccine express idiotypic determinants indicative of V(H)3 gene segment usage. J Infect Dis 178:707–716. doi:10.1086/515369. 9728539

[19] Lucas AH, Granoff DM, Mandrell RE, Connolly CC, Shan AS, Powers DC. 1997. Oligoclonality of serum immunoglobulin G antibody responses to Streptococcus pneumoniae capsular polysaccharide serotypes 6B, 14, and 23F. Infect Immun 65:5103–5109. doi:10.1128/IAI.65.12.5103-5109.1997. 9393802 PMC175735

[20] Zhong Z, Burns T, Chang Q, Carroll M, Pirofski L. 1999. Molecular and functional characteristics of a protective human monoclonal antibody to serotype 8 Streptococcus pneumoniae capsular polysaccharide. Infect Immun 67:4119–4127. doi:10.1128/IAI.67.8.4119-4127.1999. 10417182 PMC96715

[21] Russell ND, Corvalan JR, Gallo ML, Davis CG, Pirofski L. 2000. Production of protective human antipneumococcal antibodies by transgenic mice with human immunoglobulin loci. Infect Immun 68:1820–1826. doi:10.1128/iai.68.4.1820-1826.2000. 10722569 PMC97353

[22] Shaw DR, Kirkham P, Schroeder HW, Jr., Roben P, Silverman GJ. 1995. Structure-function studies of human monoclonal antibodies to pneumococcus type 3 polysaccharide. Ann N Y Acad Sci 764:370–373. doi:10.1111/j.1749-6632.1995.tb55849.x. 7486548

[23] Fletcher MA, Schmitt HJ, Syrochkina M, Sylvester G. 2014. Pneumococcal empyema and complicated pneumonias: global trends in incidence, prevalence, and serotype epidemiology. Eur J Clin Microbiol Infect Dis 33:879–910. doi:10.1007/s10096-014-2062-6. 24563274 PMC4110404

[24] Alonso M, Marimon JM, Ercibengoa M, Perez-Yarza EG, Perez-Trallero E. 2013. Dynamics of Streptococcus pneumoniae serotypes causing acute otitis media isolated from children with spontaneous middle-ear drainage over a 12-year period (1999–2010) in a region of northern Spain. PLoS One 8:e54333. doi:10.1371/journal.pone.0054333. 23349853 PMC3551958

[25] Hausdorff WP, Bryant J, Paradiso PR, Siber GR. 2000. Which pneumococcal serogroups cause the most invasive disease: implications for conjugate vaccine formulation and use, part I. Clin Infect Dis 30:100–121. doi:10.1086/313608. 10619740

[26] Magee AD, Yother J. 2001. Requirement for capsule in colonization by Streptococcus pneumoniae. Infect Immun 69:3755–3761. doi:10.1128/IAI.69.6.3755-3761.2001. 11349040 PMC98386

[27] Avery OT, Dubos R. 1931. The protective action of a specific enzyme against type III Pneumococcus infection in mice. J Exp Med 54:73–89. doi:10.1084/jem.54.1.73. 19869903 PMC2132045

[28] Middleton DR, Paschall AV, Duke JA, Avci FY. 2018. Enzymatic hydrolysis of pneumococcal capsular polysaccharide renders the bacterium vulnerable to host defense. Infect Immun 86:e00316-18. doi:10.1128/IAI.00316-18. 29866907 PMC6056847

[29] Paschall AV, Middleton DR, Wantuch PL, Avci FY. 2020. Therapeutic activity of type 3 Streptococcus pneumoniae capsule degrading enzyme Pn3Pase. Pharm Res 37:236. doi:10.1007/s11095-020-02960-3. 33140159 PMC7605875

[30] Li G, Li L, Xue C, Middleton D, Linhardt RJ, Avci FY. 2015. Profiling pneumococcal type 3-derived oligosaccharides by high resolution liquid chromatography-tandem mass spectrometry. J Chromatogr A 1397:43–51. doi:10.1016/j.chroma.2015.04.009. 25913329 PMC4426055

[31] Middleton DR, Zhang X, Wantuch PL, Ozdilek A, Liu X, LoPilato R, Gangasani N, Bridger R, Wells L, Linhardt RJ, Avci FY. 2018. Identification and characterization of the Streptococcus pneumoniae type 3 capsule-specific glycoside hydrolase of Paenibacillus species 32352. Glycobiology 28:90–99. doi:10.1093/glycob/cwx097. 29190349 PMC6192388

[32] Geno KA, Gilbert GL, Song JY, Skovsted IC, Klugman KP, Jones C, Konradsen HB, Nahm MH. 2015. Pneumococcal capsules and their types: past, present, and future. Clin Microbiol Rev 28:871–899. doi:10.1128/CMR.00024-15. 26085553 PMC4475641

[33] Lapidot R, Shea K, Yildirim I, Cabral H, Pelton S, The Massachusetts Department Of Public Health. 2020. Characteristics of serotype 3 invasive pneumococcal disease before and after universal childhood immunization with PCV13 in Massachusetts. Pathogens 9:396. doi:10.3390/pathogens9050396. PMC7281000 32455770

[34] Luck JN, Tettelin H, Orihuela CJ. 2020. Sugar-coated killer: serotype 3 pneumococcal disease. Front Cell Infect Microbiol 10:613287. doi:10.3389/fcimb.2020.613287. 33425786 PMC7786310

[35] Linley E, Bell A, Gritzfeld JF, Borrow R. 2019. Should pneumococcal serotype 3 be included in serotype-specific immunoassays? Vaccines (Basel 7):4. doi:10.3390/vaccines7010004. PMC6466091 30609868

[36] Silva-Costa C, Brito MJ, Pinho MD, Friaes A, Aguiar SI, Ramirez M, Melo-Cristino J, Portuguese Group for the Study of Streptococcal Infections, Portuguese Study Group of Invasive Pneumococcal Disease of the Pediatric Infectious Disease Society. 2018. Pediatric complicated pneumonia caused by Streptococcus pneumoniae serotype 3 in 13-valent pneumococcal conjugate vaccinees, Portugal, 2010–2015. Emerg Infect Dis 24:1307–1314. doi:10.3201/eid2407.180029. 29912700 PMC6038763

[37] Horacio AN, Lopes JP, Ramirez M, Melo-Cristino J, Portuguese Group for the Study of Streptococcal Infections. 2014. Non-invasive pneumococcal pneumonia in Portugal—serotype distribution and antimicrobial resistance. PLoS One 9:e103092. doi:10.1371/journal.pone.0103092. 25075961 PMC4116175

[38] Wijayasri S, Hillier K, Lim GH, Harris TM, Wilson SE, Deeks SL. 2019. The shifting epidemiology and serotype distribution of invasive pneumococcal disease in Ontario, Canada, 2007–2017. PLoS One 14:e0226353. doi:10.1371/journal.pone.0226353. 31834926 PMC6910703

[39] Andrews NJ, Waight PA, Burbidge P, Pearce E, Roalfe L, Zancolli M, Slack M, Ladhani SN, Miller E, Goldblatt D. 2014. Serotype-specific effectiveness and correlates of protection for the 13-valent pneumococcal conjugate vaccine: a postlicensure indirect cohort study. Lancet Infect Dis 14:839–846. doi:10.1016/S1473-3099(14)70822-9. 25042756

[40] Ye J, Ma N, Madden TL, Ostell JM. 2013. IgBLAST: an immunoglobulin variable domain sequence analysis tool. Nucleic Acids Res 41:W34–W40. doi:10.1093/nar/gkt382. 23671333 PMC3692102

[41] Asensio JL, Arda A, Canada FJ, Jimenez-Barbero J. 2013. Carbohydrate-aromatic interactions. Acc Chem Res 46:946–954. doi:10.1021/ar300024d. 22704792

[42] Spiwok V. 2017. CH/pi interactions in carbohydrate recognition. Molecules 22:1038. doi:10.3390/molecules22071038. PMC6152320 28644385

[43] Paschall AV, Middleton DR, Avci FY. 2019. Opsonophagocytic killing assay to assess immunological responses against bacterial pathogens. J Vis Exp doi:10.3791/59400. PMC7201466 31009013

[44] Roche AM, Richard AL, Rahkola JT, Janoff EN, Weiser JN. 2015. Antibody blocks acquisition of bacterial colonization through agglutination. Mucosal Immunol 8:176–185. doi:10.1038/mi.2014.55. 24962092 PMC4268183

[45] Kirkham PM, Mortari F, Newton JA, Schroeder HW. Jr., 1992. Immunoglobulin VH clan and family identity predicts variable domain structure and may influence antigen binding. EMBO J 11:603–609. doi:10.1002/j.1460-2075.1992.tb05092.x. 1537339 PMC556492

[46] Dutzler R, Wang YF, Rizkallah P, Rosenbusch JP, Schirmer T. 1996. Crystal structures of various maltooligosaccharides bound to maltoporin reveal a specific sugar translocation pathway. Structure 4:127–134. doi:10.1016/s0969-2126(96)00016-0. 8805519

[47] Robert X, Haser R, Gottschalk TE, Ratajczak F, Driguez H, Svensson B, Aghajari N. 2003. The structure of barley alpha-amylase isozyme 1 reveals a novel role of domain C in substrate recognition and binding: a pair of sugar tongs. Structure 11:973–984. doi:10.1016/S0969-2126(03)00151-5. 12906828

[48] Martin-Santamaria S, Andre S, Buzamet E, Caraballo R, Fernandez-Cureses G, Morando M, Ribeiro JP, Ramirez-Gualito K, de Pascual-Teresa B, Canada FJ, Menendez M, Ramstrom O, Jimenez-Barbero J, Solis D, Gabius HJ. 2011. Symmetric dithiodigalactoside: strategic combination of binding studies and detection of selectivity between a plant toxin and human lectins. Org Biomol Chem 9:5445–5455. doi:10.1039/c0ob01235a. 21660340

[49] Wheeler SE, Bloom JW. 2014. Toward a more complete understanding of noncovalent interactions involving aromatic rings. J Phys Chem A 118:6133–6147. doi:10.1021/jp504415p. 24937084

[50] Dill KA. 1990. Dominant forces in protein folding. Biochemistry 29:7133–7155. doi:10.1021/bi00483a001. 2207096

[51] Haji-Ghassemi O, Blackler RJ, Martin Young N, Evans SV. 2015. Antibody recognition of carbohydrate epitopes. Glycobiology 25:920–952. doi:10.1093/glycob/cwv037. 26033938

[52] Jeffrey PD, Bajorath J, Chang CY, Yelton D, Hellstrom I, Hellstrom KE, Sheriff S. 1995. The x-ray structure of an anti-tumour antibody in complex with antigen. Nat Struct Biol 2:466–471. doi:10.1038/nsb0695-466. 7664109

[53] Villeneuve S, Souchon H, Riottot MM, Mazie JC, Lei P, Glaudemans CP, Kovac P, Fournier JM, Alzari PM. 2000. Crystal structure of an anti-carbohydrate antibody directed against Vibrio cholerae O1 in complex with antigen: molecular basis for serotype specificity. Proc Natl Acad Sci U S A 97:8433–8438. doi:10.1073/pnas.060022997. 10880560 PMC26965

[54] Brooks CL, Muller-Loennies S, Brade L, Kosma P, Hirama T, MacKenzie CR, Brade H, Evans SV. 2008. Exploration of specificity in germline monoclonal antibody recognition of a range of natural and synthetic epitopes. J Mol Biol 377:450–468. doi:10.1016/j.jmb.2008.01.018. 18272175

[55] Nguyen HP, Seto NO, MacKenzie CR, Brade L, Kosma P, Brade H, Evans SV. 2003. Germline antibody recognition of distinct carbohydrate epitopes. Nat Struct Biol 10:1019–1025. doi:10.1038/nsb1014. 14625588

[56] Yano M, Pirofski LA. 2011. Characterization of gene use and efficacy of mouse monoclonal antibodies to Streptococcus pneumoniae serotype 8. Clin Vaccine Immunol 18:59–66. doi:10.1128/CVI.00368-10. 21068211 PMC3019784

[57] Wong SE, Sellers BD, Jacobson MP. 2011. Effects of somatic mutations on CDR loop flexibility during affinity maturation. Proteins 79:821–829. doi:10.1002/prot.22920. 21287614

[58] Avci FY. 2013. Novel strategies for development of next-generation glycoconjugate vaccines. Curr Top Med Chem 13:2535–2540. doi:10.2174/15680266113136660180. 24066893

[59] Wantuch PL, Avci FY. 2018. Current status and future directions of invasive pneumococcal diseases and prophylactic approaches to control them. Hum Vaccin Immunother 14:2303–2309. doi:10.1080/21645515.2018.1470726. 29757699 PMC6183136

[60] Wantuch PL, Avci FY. 2019. Invasive pneumococcal disease in relation to vaccine type serotypes. Hum Vaccin Immunother 15:874–875. doi:10.1080/21645515.2018.1564444. 30668209 PMC6605867

[61] Middleton DR, Sun L, Paschall AV, Avci FY. 2017. T cell-mediated humoral immune responses to type 3 capsular polysaccharide of Streptococcus pneumoniae. J Immunol 199:598–603. doi:10.4049/jimmunol.1700026. 28566369 PMC5515494

[62] Avci FY, Li X, Tsuji M, Kasper DL. 2011. A mechanism for glycoconjugate vaccine activation of the adaptive immune system and its implications for vaccine design. Nat Med 17:1602–1609. doi:10.1038/nm.2535. 22101769 PMC3482454

[63] Avci FY, Li X, Tsuji M, Kasper DL. 2012. Isolation of carbohydrate-specific CD4(+) T cell clones from mice after stimulation by two model glycoconjugate vaccines. Nat Protoc 7:2180–2192. doi:10.1038/nprot.2012.138. 23196974 PMC4133772

[64] Barbas CF, Burton DR, Scott JK, Silverman GJ. 2004. Phage display: a laboratory manual. Cold Spring Harbor Laboratory Press, Cold Spring Harbor, NY.

[65] Burton RL, Nahm MH. 2012. Development of a fourfold multiplexed opsonophagocytosis assay for pneumococcal antibodies against additional serotypes and discovery of serological subtypes in Streptococcus pneumoniae serotype 20. Clin Vaccine Immunol 19:835–841. doi:10.1128/CVI.00086-12. 22518015 PMC3370448

[66] Habets MN, van Selm S, van der Gaast-de Jongh CE, Diavatopoulos DA, de Jonge MI. 2017. A novel flow cytometry-based assay for the quantification of antibody-dependent pneumococcal agglutination. PLoS One 12:e0170884. doi:10.1371/journal.pone.0170884. 28288168 PMC5347990

[67] Mitsi E, Roche AM, Reine J, Zangari T, Owugha JT, Pennington SH, Gritzfeld JF, Wright AD, Collins AM, van Selm S, de Jonge MI, Gordon SB, Weiser JN, Ferreira DM. 2017. Agglutination by anti-capsular polysaccharide antibody is associated with protection against experimental human pneumococcal carriage. Mucosal Immunol 10:385–394. doi:10.1038/mi.2016.71. 27579859 PMC5332540

