# Supplementary material for: A Structural Model for the Ligand Binding of Pneumococcal Serotype 3 Capsular Polysaccharide-Specific Protective Antibodies
Source: mBio. 2021 Jun 1;12(3):e00800-21. doi: 10.1128/mBio.00800-21 (PMC8262990; doi:10.1128/mBio.00800-21)
Supplement: FIG S1 [file mbio.00800-21-sf001.pdf]

	V-D Junction	D_H	D-J Junction
**Germline**		GTTACGA	
**5.6**	AGGGTAGTA	GTAACGA	AAGGGGGG
**25.1**	AGGCTGGTT	GTTACGA	AAGGGGGG
**64.4**	AGGGTAGTT	GTTACGA	GAGGGGGG

	V-D Junction	D_H	D-J Junction
**Germline**		TTACGAC	
**75.3**	GGAAATT	TTACGAC	GGGGG
**90.1**	GGAAATT	TTACGAC	GGGGG
