# Supplementary material for: A Structural Model for the Ligand Binding of Pneumococcal Serotype 3 Capsular Polysaccharide-Specific Protective Antibodies
Source: mBio. 2021 Jun 1;12(3):e00800-21. doi: 10.1128/mBio.00800-21 (PMC8262990; doi:10.1128/mBio.00800-21)
Supplement: FIG S2 [file mbio.00800-21-sf002.pdf]

```
            1         11        21    26     35    41           51      63
Germline    EVKLVESGGGLVKPGGSLKLSCAAS GFTFSSYA MSWVRQTPEKRLEWVAS ISSGGST YYP
5.6 H       EVKLVESGGGLVKPGGSLKLSCAAS GFTFSNYA MSWVRQTPEKRLEWVAS IIIGGNT YYP
64.4 H      EVKLEESGGDLVKPGGSLKLSCAAS GFTFSSYA MSWVRQTPEKRLEWVAS IISGGRT YYR
25.1 H      EVKLEESGGGLVKPGGSLKLSCAAS GFTFSNYA MSWVRQTPEKRLEWVAS IIIGGNT YYP
                                        CDR1                     CDR2

            69 71        81        91        101       111          121
Germline    DSVKGRFTISRDNARNILYLQMSSLRSEDTAMYYC AR-VVV----AMDY WGQGTSVTVSS
5.6 H       DSVKGRFTISRDNARNILYLQMSSLRSEDTAMYYC TRRVVVTKGGAMDY WGQGTSVTVSS
64.4 H      DSVKGRFTISRDNDRNILFLQMSSLRSEDTAMYFC ARRVVVTRGGAMDY WGQGTSVTVSS
25.1 H      NSVKGRFTISRDNARNILYLQMSSLRSEDTAVYYC TRRLVVTKGGAMDY WGQGTSVTVSS
                                                     CDR3

            1         11        21       37   41           51   66   71
Germline    QIVLTQSPAIMSASPGEKVTMTCSAS SSVSY MYWYQQKPGSSPRLLIY DTS NLASGVPVR
5.6 L       QIVLTQSPAIMSASPGEKVTMTCSAS SSVSY MYWYQQKPGSSPRLLIY DTS NLASGVPVR
64.4 L      ELVLTQSPAIMSASPGEKVTMTCSAS SSVSY MYWYQQKPGSSPRLLIY DTS NLASGVPVR
25.1 L      ELVLTQSPALMSASPGEKVTMTCSAI SSVDY MYWYQQKPGSSPRLLIY DTS NLASGVPVR
                                        CDR1                    CDR2

            76    83        91             101       111       121
Germline    FSGSGSGTSYSLTISRMEAEDAATYYC QQWSSYPFT FGSGTKLEIK
5.6 L       FSGSGSGTSYSLTISRMEAEDAATYYC QQWSTYPFT FGSGTKLEIK
64.4 L      FSGSGSVTSYSLTISRMEAEDAATYYC QQWSSYPFT FGSGTKLEIK
25.1 L      FGGSGSGTSYSLTISRMEAEDAATYYC QQWSYYPFT FGSGTKLEIK
                                          CDR3

            1         11        21       35     41          51       62
Germline    QIQLVQSGPELKKPGETVKISCKAS GYTFTNYG MNWVKQAPGKGLKWMGW INTYTGEP TY
75.3 H      QIQLVQSGPELKKPGETVKISCKAS GYTFTNFG MNWVKQAPGKGLKWMGW IHTYTGES TY
90.1 H      QVQVEQSGPELKKPGETVKISCKAS GYTFTNFG MNWVKQAPGNGLKWMGW INSYTGEP TY
                                        CDR1                        CDR2

                 74        81        91        101       111          121
Germline    ADDFKGRFAFSLETSASTAYLQINNLKNEDTATYFC ARGNFTTGAWFAY WGQGTLVTVSA
75.3 H      ADDFKRRFAFSLEPSASTAFLQINNLKNEDTATYFC VRGNFTTGAWFTF WGQGSLVTVSA
90.1 H      ADDFKGRFAFSLETSASTAFLQINNLKNEDTATYFC VRGNFTTGAWFTF WGQGTLVTVST
                                                      CDR3

            1         11        21           34       41           51   65
Germline    DAVMTQTPLSLPVSLGDQASISCRSS QSLENSNGNTY LNWYLQKPGQSPQLLIY RVS NRF
75.3 L      DVVMTQTPLSLPVSLGDQASISCRSS QSLENSNGNSY LNWYFQKPGQSPQLLIY KVS NRF
90.1 L      ELVMTQTPLSLPVSLGDQASISCRSS QNLENSNGNSY LNWYLQKPGQSPQLLIY KVS NRF
                                          CDR1                       CDR2

                74        83       91        101       111        121
Germline    SGVLDRFSGSGSGTDFTLKISRVEAEDLGVYFC LQVTHVPWT FGGGTKLEIK
75.3 L      SGVQDRFSGSGSGTEFTLKISRVEAEDLGVYFC LQVTHVPWT FGGGTKLEIK
90.1 L      SGVQDRFSGSGSGTEFTLKISRVEAEDLGVYFC LQVTHVPWT FGGGTKLEIK
                                                CDR3
```
