# Supplementary material for: A Structural Model for the Ligand Binding of Pneumococcal Serotype 3 Capsular Polysaccharide-Specific Protective Antibodies
Source: mBio. 2021 Jun 1;12(3):e00800-21. doi: 10.1128/mBio.00800-21 (PMC8262990; doi:10.1128/mBio.00800-21)
Supplement: FIG S3 [file mbio.00800-21-sf003.pdf]

	1 … 11 … 21 … 26 … 35 … 41 … 51 … 63
Human V_H 3-69-1	EVQLVESGGGLVKPGGSLRLSCAAS GFTFSDYY MNWVRQAPGKGLEWVSS ISSSSTI YYA
5.6 V_H	EVKLEESGGGLVKPGGSLKLSCAAS GFTFSNYA MSWVRQTPEKRLEWVAS IIIGGNT YYP
	CDR1 … CDR2

	69 71 … 81 … 91 … 101
Human V_H 3-69-1	DSVKGRFTISRDNAKNSLYLQMNSLRAEDTAVYYC AR
5.6 V_H	DSVKGRFTISRDNARNILYLQMSSLRSEDTAMYYC TR
	CDR3
