# Supplementary material for: A Structural Model for the Ligand Binding of Pneumococcal Serotype 3 Capsular Polysaccharide-Specific Protective Antibodies
Source: mBio. 2021 Jun 1;12(3):e00800-21. doi: 10.1128/mBio.00800-21 (PMC8262990; doi:10.1128/mBio.00800-21)
Supplement: TABLE S1 [file mbio.00800-21-st001.docx]

**Table S1.** Data collection and refinement statistics for 5.6 Fab - Pn3 hexasaccharide. Statistics for the highest-resolution shell are shown in parentheses.

| **Wavelength** | 1.000 Å |
| --- | --- |
| **Resolution range** | 38.1 - 2.3 (2.382 - 2.3) |
| **Space group** | C 2 2 21 |
| **Unit cell** | 49.516 134.302 133.119 90 90 90 |
| **Total reflections** | 262439 (27401) |
| **Unique reflections** | 20196 (2008) |
| **Multiplicity** | 13.0 (13.6) |
| **Completeness (%)** | 99.94 (100.00) |
| **Mean I/sigma(I)** | 16.36 (2.59) |
| **Wilson B-factor** | 46.02 |
| **R-merge** | 0.1071 (1.134) |
| **R-meas** | 0.1116 (1.179) |
| **R-pim** | 0.03096 (0.3179) |
| **CC1/2** | 0.999 (0.835) |
| **CC*** | 1 (0.954) |
| **Reflections used in refinement** | 20191 (2008) |
| **Reflections used for R-free** | 1999 (199) |
| **R-work** | 0.1854 (0.2853) |
| **R-free** | 0.2243 (0.3598) |
| **CC (work)** | 0.967 (0.862) |
| **CC (free)** | 0.958 (0.740) |
| **Number of non-hydrogen atoms** | 3331 |
| **Macromolecules** | 3224 |
| **Ligands** | 35 |
| **Solvent** | 72 |
| **Protein residues** | 426 |
| **RMS (bonds)** | 0.011 |
| **RMS (angles)** | 1.16 |
| **Ramachandran favored (%)** | 96.19 |
| **Ramachandran allowed (%)** | 3.81 |
| **Ramachandran outliers (%)** | 0 |
| **Rotamer outliers (%)** | 0.27 |
| **Clashscore** | 8.15 |
| **Average B-factor** | 48.29 |
| **Macromolecules** | 48.28 |
| **Ligands** | 48.93 |
| **Solvent** | 48.29 |
